# Effect of impregnation strategy on structural characteristics of Ce–Mn/Al_2_O_3_ and its catalytic ozonation of benzoic acid†

**DOI:** 10.1039/d4ra06148a

**Published:** 2024-09-30

**Authors:** Shengjuan Shao, Ting Cheng, Yifan Cheng, Bingxin Chen

**Affiliations:** a Department of Chemistry and Chemical Engineering, Taiyuan Institute of Technology Taiyuan 030008 China shaosj_tit@163.com; b School of Chemistry and Chemical Engineering, North University of China Taiyuan Shanxi 030051 China

## Abstract

Ce–Mn binary oxides supported on Al_2_O_3_ (Ce–Mn/Al_2_O_3_), with enhanced activity and stability for catalytic ozonation of benzoic acid, were synthesized using a facile impregnation method. The competitive synergetic effects between cerium and manganese significantly influenced the structural characteristics and catalytic performance of the catalysts depending on the impregnation sequence. Catalysts prepared *via* the one-step impregnation process exhibited a higher concentration of homogeneous Ce^3+^ species on the catalyst surface. This led to an increase in surface oxygen vacancies, thereby enhancing catalytic activity. In contrast, the two-step impregnation process resulted in fewer oxygen vacancies due to reduced competitive effects between cerium and manganese. Overall, the optimized Ce–Mn/Al_2_O_3_ catalysts demonstrated improved catalytic performance in ozonation reactions, highlighting the importance of impregnation method and calcination conditions in tailoring catalyst properties for enhanced activity and stability. Oxygen vacancies play a crucial role as active sites for ozone adsorption and dissociation into *O_2_ and *O, facilitated by the reduction of Mn^4+^ to Mn^3+^ and the oxidation of Ce^3+^ to Ce^4+^. This process forms an electron closed loop that maintains electron balance. The synergistic interactions between cerium and manganese enable rapid electron transfer between Ce^4+^ and Mn^3+^, facilitating the regeneration of Ce^3+^ and Mn^4+^. Due to the increase of the dual redox conjugate pairs and the surface reactive oxygen species, the catalytic ozonation activity and stability of Ce–Mn/Al_2_O_3_ was enhanced.

## Abstract 2

Ce–Mn binary oxides supported on Al_2_O_3_ (Ce–Mn/Al_2_O_3_), with enhanced activity and stability for catalytic ozonation of benzoic acid, were synthesized using a facile impregnation method.

## Graphical abstract

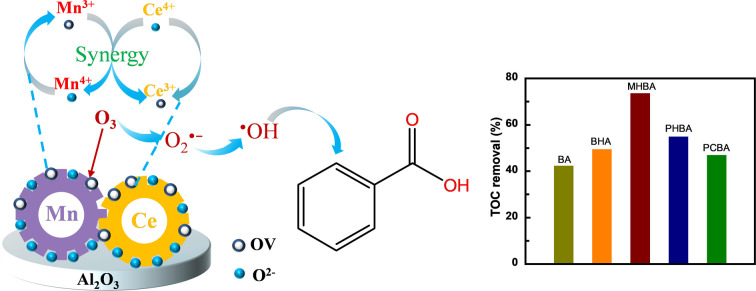

## 1. Introduction

Heterogeneous catalytic ozonation is considered to be one of the effective methods for the refractory organic pollutants in water.^1^ Dhandapani *et al.*^2^ conducted a comparative study on the catalytic ozonation capabilities of various metal oxides (Ag, Mn, Fe, Cu, Mg, Ce, Co, Cr, Ni, and V) supported on Al_2_O_3_. Among these, MnO_2_/Al_2_O_3_ exhibited superior catalytic ozonation performance attributed to its excellent reducibility and the characteristics of p-type oxides facilitated the decomposition of O_3_ into superoxide (˙O_2_^−^), enhancing the overall efficiency. Faria *et al.*^3^ prepared mixed oxides-supported catalysts using the transition metal Mn and rare earth element Ce for catalytic ozonation of sulfanilic acid and aniline. The Ce–Mn mixed oxides demonstrated larger specific surface areas and stronger redox capacities compared to their single oxide counterparts. This resulted in significant improvements in the mineralization of sulfanilic acid and aniline. The enhanced catalytic performance was primarily attributed to improved lattice oxygen mobility and increased surface reactive oxygen species, highlighting synergistic interactions between Ce and Mn.^4–6^ Previous research has also indicated that the enhanced ozonation efficiency observed in Ce-containing catalysts is largely due to increased concentrations of Ce(iii) and oxygen vacancies.^7–9^ As is known to all, CeO_2_ possesses a unique 4f orbital that is incompletely filled with electrons, enabling it to serve as an electron reservoir facilitating rapid conversion between Ce^3+^ and Ce^4+^. This characteristic endows CeO_2_ with high oxygen storage capacity and efficient oxygen transfer abilities. These findings underscore the importance of understanding metal oxide interactions and their impact on catalytic processes for water treatment applications.

Our previous work has also demonstrated that the redox pairs and oxygen vacancy had a synergetic effect on the catalytic ozonation of nitrobenzene wastewater by Ce–Mn/Al_2_O_3_.^10^ However, the effects of preparation methods on the structural characteristics of Ce–Mn/Al_2_O_3_, especially the effects of impregnation sequence were not elucidated in our previous work. Zhu *et al.*^11^ investigated the impact of impregnation sequence on the hydrothermal stability and NH_3_-SCR activity of Ce–Nb/SnO_2_ catalysts. They found that catalysts impregnated first with Nb followed by Ce exhibited enhanced synergy between Nb and Sn, showcasing the strongest redox capability while also demonstrating superior structural stability and NH_3_-SCR activity. In a similar vein, Ozcan *et al.*^12^ compared the catalytic performance and stability of alumina-supported Co, W, Zr catalysts prepared using sequential impregnation and co-impregnation methods for biofuel production. Their results highlighted that the catalysts prepared *via* co-impregnation showed the highest bio-oil conversion rate and selectivity. These studies underscore the significant impact of impregnation methods, particularly impregnation sequence, on catalyst structure and catalytic activity. Further research in this area holds promise for optimizing catalyst design and performance in heterogeneous catalytic ozonation processes.

Benzoic acid (BA) is an aromatic organic compound widely employed as a food preservative in beverages, juices, cold foods, soy sauce, vinegar, and canned foods. It acts as a bacteriostatic agent, effectively inhibiting the growth of bacteria, molds, and fungi. Due to its resistance to biodegradation by traditional microbial methods,^13^ effective treatment methods for benzoic acid wastewater include photocatalytic oxidation,^14,15^ electrochemical oxidation,^16,17^ and heterogeneous catalytic ozonation.^18–21^ Qin *et al.*^18^ investigated the catalytic performance of cerium oxide supported on N, S-doped activated carbon (Ce/ACNS) for the ozonation of benzoic acid. They attributed the superior activity of Ce/ACNS to increased surface electron density of N and S atoms, along with higher Ce(iii) content on the surface. Fijołek *et al.*^19^ studied CePO_4_, CeO_2_, and bifunctional CePO_4_/CeO_2_ nanocomposites for catalytic ozonation of benzoic acid in the presence of chlorides. Among these, CePO_4_/CeO_2_ exhibited the best ozonation recovery performance, and the improved reactivity was attributed to the synergistic interaction between CePO_4_ and CeO_2_ in the bifunctional nanocomposite. Franco Peláez *et al.*^20^ utilized CeO_2_ thin films to catalyze the ozonation of benzoic acid. They found that the oxidation of Ce^3+^ contributed to the formation of reactive oxygen species, facilitating high mineralization of benzoic acid. These findings underscore the diverse applications and effective mechanisms of cerium-based catalysts in advancing the treatment of benzoic acid wastewater through ozonation processes.

In this study, we evaluated the catalytic ozonation efficiency of Ce–Mn/Al_2_O_3_ using benzoic acid as the target pollutant. The main objectives were threefold: (1) to investigate the impact of different preparation methods, particularly the impregnation sequence, on the structural characteristics and catalytic properties of Ce–Mn/Al_2_O_3_. (2) To assess the catalytic activity and stability of Ce–Mn/Al_2_O_3_ in the degradation of benzoic acid through catalytic ozonation. (3) To elucidate the synergistic interactions between Ce and Mn binary oxides, focusing on how they enhance the overall catalytic performance. These objectives aimed to deepen our understanding of Ce–Mn/Al_2_O_3_ as a catalyst and its potential application in environmental remediation processes involving ozonation.

## 2. Experimental procedure

### 2.1 Catalyst preparation

The catalysts were prepared according to the impregnation methods including the one-step impregnation and two-step impregnation method. Ce–Mn/Al_2_O_3_ catalysts were synthesized with varying amounts of Mn and Ce at different Mn to Ce molar ratios (7 : 3, 3 : 1, 1 : 1, and 2 : 1).

For instance, the Ce–Mn/Al_2_O_3_ catalyst prepared *via* the one-step impregnation method had a total (Mn + Ce) content of 8.0 wt% and a Mn/Ce molar ratio of 7 : 3. Specifically, 0.34 g of Mn(CH_3_COO)_2_·4H_2_O and 0.26 g of Ce(NO_3_)_3_·6H_2_O (supplied by Shanghai Maclin Biochemical Technology Co., Ltd) were dissolved in 5 mL of deionized water. 2.0 g of γ-Al_2_O_3_ (∼2 mm) were added and ultrasonically impregnated for 60 minutes. The impregnated samples were then dried at 100 °C for 12 hours and calcined at temperatures ranging from 400 to 700 °C for 4 hours to obtain the Ce–Mn/Al_2_O_3_ catalyst.

For the two-step impregnation method, Ce–Mn(F)/Al_2_O_3_ was prepared by first impregnating Mn and then impregnating Ce; Ce(F)–Mn/Al_2_O_3_ was prepared by first impregnating Ce and then impregnating Mn. Mn/Al_2_O_3_ and Ce/Al_2_O_3_ were also prepared by the same method with the single metal content of 8.0 wt%.

### 2.2 Catalyst characterization

Transmission electron microscopy (TEM) images were acquired using an FEI Tecnai F20. X-ray diffraction (XRD) patterns were carried out on the Rigaku SmartLab powder diffractometer using a CuKα radiation source (*λ* = 1.541 Å) with the scanning range of 10–80°. The nitrogen adsorption/desorption Brunauer–Emmett–Teller (BET) tests were performed using the Micromeritics TriStar II surface area analyzer, and the average pore diameter of catalysts were calculated by the Barrett–Joyner–Halenda (BJH) method. X-ray photoelectron spectroscopy (XPS) spectra were determined on the Thermofisher K-Alpha spectrometer using an AlKα radiation source of 1487 eV with the spot size of 400 μm, and the binding energies were calibrated with reference to the C 1s line at 284.8 eV. Electron paramagnetic resonance (EPR) tests were performed on the Bruker A300 spectrometer at X-band (9.5 GHz) at 77 K. Raman spectra were obtained using a Horiba Scientific LabRAM HR Evolution spectrometer equipped with a laser emitting at an excitation wavelength of 514 nm.

### 2.3 Catalytic ozonation process

All experiments were performed in a glass column reactor with an effective volume of 1000 mL. Ozone was generated by the 3S–T3 ozone generator and dispersed into microbubbles by an aeration stick (Fig. S1†). Ozone concentration *C*_O_3__ was maintained at 7.5 mg L^−1^ with the flow rate *V*_O_3__ of 0.5 L min^−1^. The concentration of benzoic acid wastewater (*c*_BA_) was 50 mg L^−1^ which was analyzed by the TU-1950 UV-Vis spectrophotometer at the wavelength of 225 nm. The total organic carbon (TOC) was analyzed using the Aurora l030C TOC analyzer. The degradation rate of benzoic acid was estimated using eqn (1).1
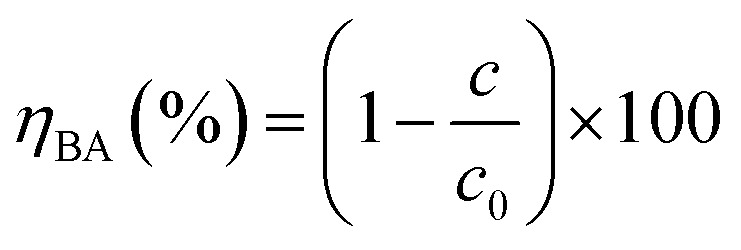
where *η*_BA_ (%) is defined as the degradation rate of benzoic acid, *c*_0_ and *c* (mg L^−1^) are the concentration of benzoic acid at the initial and sampling time, respectively.

### 2.4 Methods of theoretical calculation

All calculations were performed using the DMol3 (Materials Studio 2018) based on the density functional theory (DFT).^22^ The exchange and correlation interactions were calculated by generalized gradient approximation non-empirical function Perdew-Wang (GGA-PW91), the core electrons were calculated by the DFT semi-core pseudopotentials (DSPP) method, and the valence electrons were calculated by double-numerical plus polarization (DNP) function. The *K* point grid of Brillouin zone integration was set to 2 × 2 × *l*, the cutoff radius was set to 4.2 Å, the convergence tolerance of self-consistent iteration was set to 2.0 × 10^−6^, and the convergence accuracy of energy, maximum force and maximum displacement was set to 1.0 × 10^−5^ Ha, 0.002 Ha Å^−1^ and 0.005 Å, respectively. A four-layer periodic slab model containing twelve γ-Al_2_O_3_ units was obtained by cutting the γ-Al_2_O_3_ bulk along the (110) surface, fixing the bottom two layers and allowing the upper two layers to relax^23^ and a 12 Å vacuum layer was added to the (110) surface along the *z* axis. A supported model of Ce–Mn cluster on Al_2_O_3_(110) surface (Ce–Mn/Al_2_O_3_(110)) was established by geometric configuration optimization.

The formation energy of oxygen vacancy on the surface of Ce–Mn/Al_2_O_3_(110) was calculated as follows:*E*_OV_ = *E*_Ce–Mn/Al_2_O_3_(110)–OV_ + 1/2*E*_O_2__ − *E*_Ce–Mn/Al_2_O_3_(110)_

The adsorption energy of O_3_ at the oxygen vacancy of Ce–Mn/Al_2_O_3_(110) was as follows:*E*_ads_ = *E*_Ce–Mn/Al_2_O_3_(110)–O_3__ − *E*_O_3__ − *E*_Ce–Mn/Al_2_O_3_(110)–OV_in which *E*_Ce–Mn/Al_2_O_3_(110)–OV_ is the total energy of the Ce–Mn cluster supported on Al_2_O_3_(110) surface with the oxygen vacancy; *E*_Ce–Mn/Al_2_O_3_(110)_ is the total energy of the interacting system of the Ce–Mn cluster with the Al_2_O_3_(110) surface; *E*_Ce–Mn/Al_2_O_3_(110)–O_3__ is the total energy of the adsorption configuration of O_3_ on Ce–Mn/Al_2_O_3_(110) surface with oxygen vacancy; and *E*_O_3_/O_2__ is the energy of the isolated O_3_/O_2_ molecules.

## 3. Results and discussion

### 3.1 TEM analysis

**Fig. 1:**
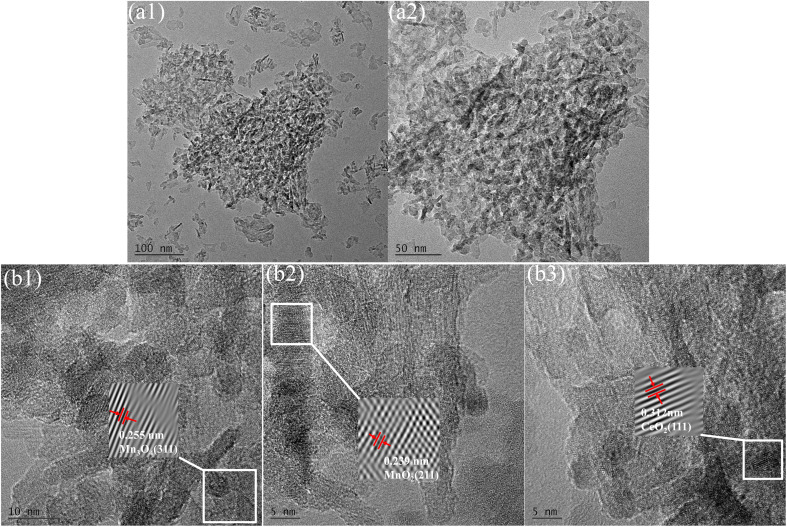
TEM images (a1 and a2) and HRTEM images (b1–b3) of Ce–Mn/γ-Al_2_O_3_.

Cerium and manganese oxides were successfully loaded onto the surface of γ-Al_2_O_3_, forming spherical (as shown in Fig. 1(a1 and a2)) and short rod-like structures. HRTEM lattice fringe images (Fig. 1(b1–b3)) further revealed specific crystal planes of cerium and manganese oxides. The lattice spacings of 0.255 nm corresponded to the (311) planes of Mn_3_O_4_ (JCPDS no. 13-0162). It has been verified that when manganese acetate is used as a precursor, MnO_*X*_ is mainly the Mn_3_O_4_ phase.^24^ Lattice spacings of 0.239 nm corresponded to the (211) planes of α-MnO_2_ (JCPDS no. 44-0141), while lattice spacings of 0.312 nm corresponded to the (111) planes of fluorite-type CeO_2_ (JCPDS no. 34-0394), indicating the presence of MnO_2_ and CeO_2_ on the catalyst surface.

### 3.2 Effect of catalyst preparation parameters on catalytic activity of Ce–Mn/Al_2_O_3_

**Fig. 2:**
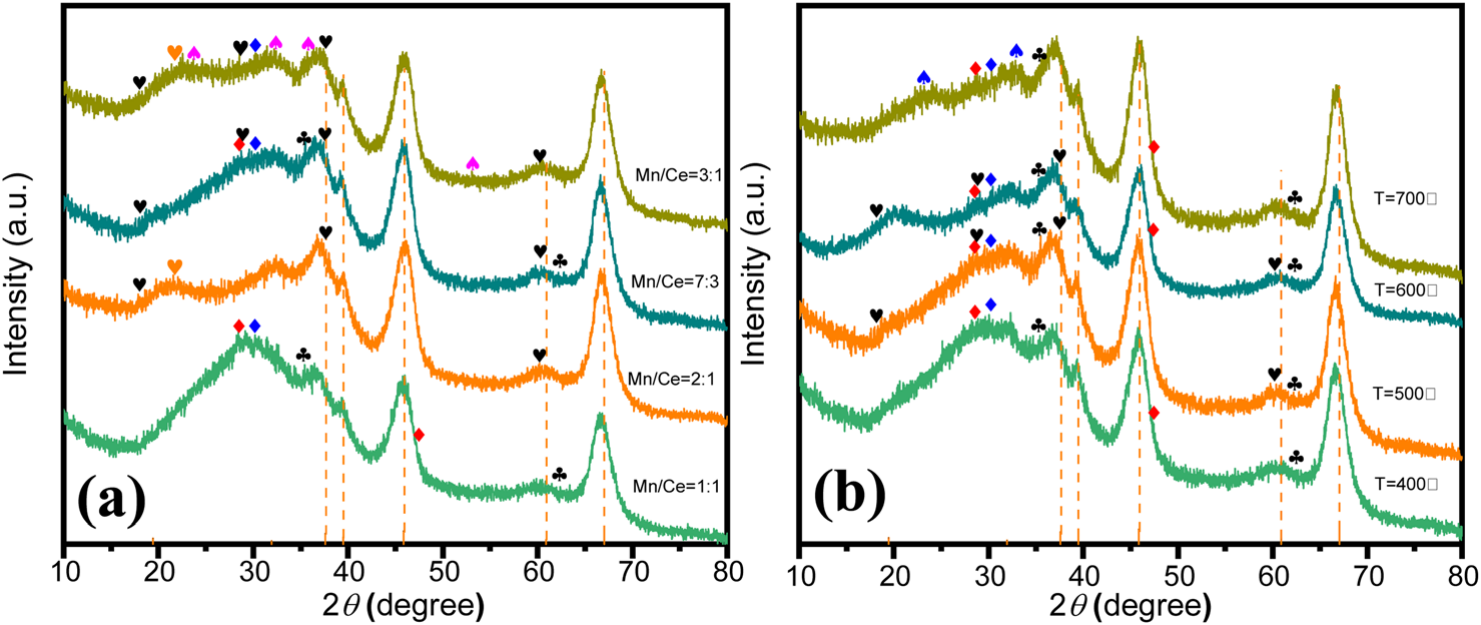
XRD patterns of Ce–Mn/Al_2_O_3_ at different molar ratios of Mn/Ce (a) and different calcination temperatures (b). (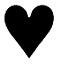 α-MnO_2_, 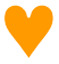 Ramsdellite, 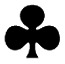 Mn_3_O_4_, 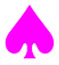 Mn_2_O_3_, 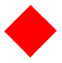 CeO_2_, 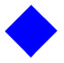 Ce_2_O_3_, 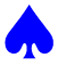 bixbyite α-Mn_2_O_3_).

The structural composition of the catalyst significantly influences its performance, particularly in relation to the molar ratio of metal precursors. Fig. 2(a) illustrates the structural and phase characteristics of synthesized Ce–Mn/Al_2_O_3_ catalysts at varying Mn/Ce molar ratios, analyzed *via* X-ray diffraction. In all samples, diffraction peaks (indicated by dashed lines) at approximately 37.60°, 39.49°, 45.86°, and 67.03° are attributed to the Al_2_O_3_ phase (JCPDS no. 10-0425). For catalysts with Mn/Ce ratios of 1 : 1 and 2 : 1, the predominant crystalline phase of manganese oxides is Mn_3_O_4_ (JCPDS no. 13-0162). With increasing Mn content, additional diffraction peaks associated with α-MnO_2_ emerge at 18.11°, 28.84°, 37.52°, and 60.27° (JCPDS no. 44-0141) in catalysts with Mn/Ce ratios of 3 : 1 and 7 : 3, which is beneficial to improve the catalytic ozonation activity due to the best redox ability of Mn^4+^ species.^25^ Ramsdellite-type MnO_2_ is detected in catalysts with Mn/Ce ratios of 2 : 1 and 3 : 1. Additionally, diffraction peaks corresponding to Mn_2_O_3_ (JCPDS no. 33-0900) are observed in the Mn/Ce = 3 : 1 catalyst, indicating the presence of Mn^3+^ with oxygen vacancies.^26^ The primary crystalline phase of cerium oxides is cerianite-type CeO_2_ (JCPDS no. 34-0394), particularly evident in the Mn/Ce = 1 : 1 catalyst. A reduction in Ce content eliminates CeO_2_ diffraction peaks, leaving only Ce_2_O_3_ (JCPDS no. 23-1048). The presence of Ce^3+^ indicates the existence of oxygen vacancies, which enhance catalytic ozonation activity.^27^ Notably, no distinct CeO_*X*_ diffraction peaks are detected in the Mn/Ce = 2 : 1 catalyst, whereas both Ce_2_O_3_ and MnO_2_ peaks are evident in Mn/Ce = 7 : 3 catalysts, indicating potentially superior catalytic activity in subsequent ozonation processes. The catalytic performances of the catalysts follow the order Mn/Ce = 7 : 3 > 3 : 1 > 1 : 1 > 2 : 1 (Fig. S2†), suggesting optimal synergistic effects between Mn and Ce at a molar ratio of 7 : 3.

Appropriate calcination temperature is also important for the catalytic performance of catalysts. Too low calcination temperatures may lead to incomplete calcination of catalyst precursor, resulting in insufficient dispersion and formation of active sites, whereas excessively high temperatures can cause sintering of active sites.^28^ For instance, at a calcination temperature of 400 °C, cerium oxides mainly consist of CeO_2_ and Ce_2_O_3_, while manganese oxides are primarily Mn_3_O_4_. Upon increasing the calcination temperature to 600 °C, diffraction peaks associated with α-MnO_2_ (JCPDS no. 44-0141) begin to appear. Comparatively, the intensity of Ce_2_O_3_ diffraction peaks increases in the catalyst calcined at 600 °C compared to that at 500 °C, indicating higher Ce^3+^ content, which makes for the improvement of catalytic ozonation activity. However, at 700 °C, the diffraction peaks of MnO_2_ disappear, replaced by bixbyite α-Mn_2_O_3_.^29^ The absence of Mn^4+^ results in decreased catalytic activity. Therefore, the catalytic performances of catalysts rank as follows: 600 °C > 500 °C > 400 °C > 700 °C, suggesting that the optimal calcination temperature is 600 °C.

**Fig. 3:**
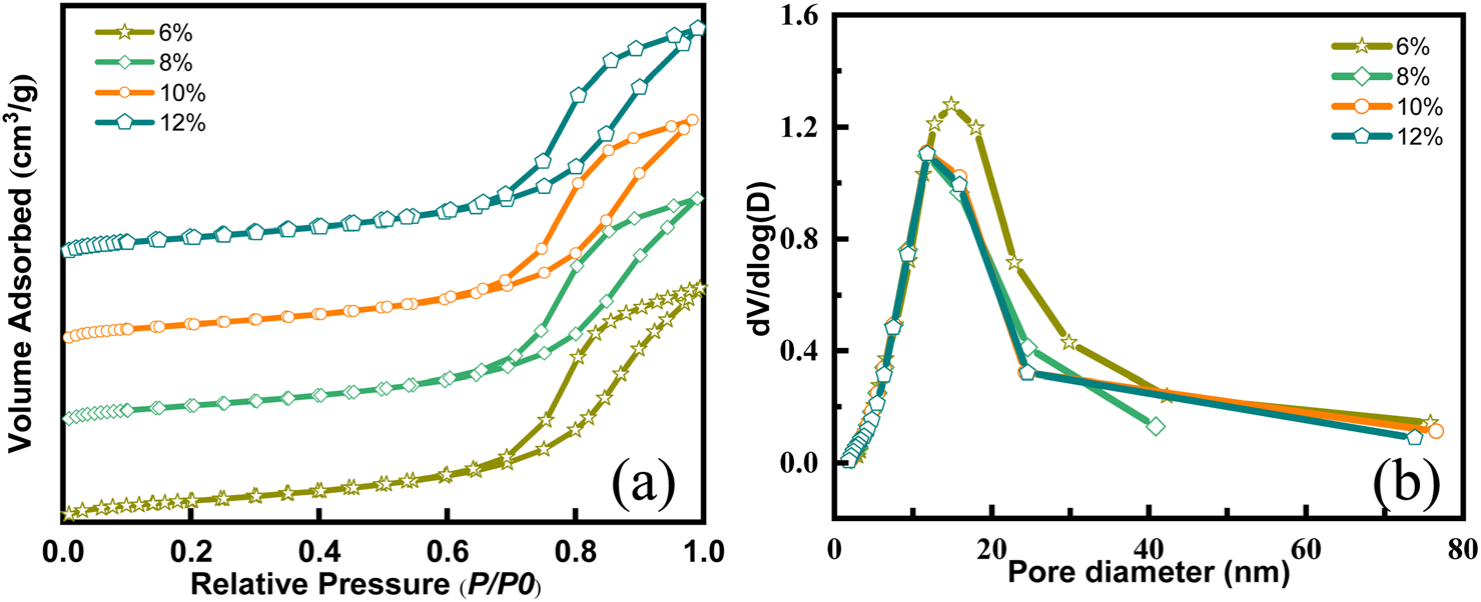
N_2_ adsorption and desorption isotherms (a) and pore size distribution curves (b) of Ce–Mn/Al_2_O_3_ with different metal loadings.

The textural properties of catalysts, such as specific surface area and pore structure, significantly influence their catalytic performance, which correlates closely with metal loadings. Insufficient loadings result in fewer active sites, whereas excessive ones can lead to surface clustering, thereby reducing catalytic ozonation performance.^28^ As depicted in Fig. 3, the adsorption–desorption isotherms of Ce–Mn/Al_2_O_3_ synthesized with varying metal loadings exhibit similar type IV isotherms with a typical H3 hysteresis loop, albeit with slight alterations in pore distribution. Metal loadings on γ-Al_2_O_3_ have been observed to decrease specific surface area (*S*_BET_) and pore volume (*V*_p_) while increasing pore diameter (*D*_a_) (Table S1†). It's noteworthy that there is minimal change in pore volume as metal loadings increase from 8% to 12%. This indicates that with the increase in metal loadings, more metal oxides are deposited onto the support surface. This phenomenon is particularly pronounced in the catalyst with %_L_ = 12%, where the increase in *S*_BET_ can be attributed precisely to the presence of metal oxides supported on the γ-Al_2_O_3_ surface. The catalytic performances of the catalysts rank as follows: %_L_ = 8% > 12% > 10% > 6%, suggesting that a metal loading of 8% is optimal.

### 3.3 Effect of impregnation sequence on catalytic ozonation activity of Ce–Mn/Al_2_O_3_

#### 3.3.1 XRD patterns

**Fig. 4:**
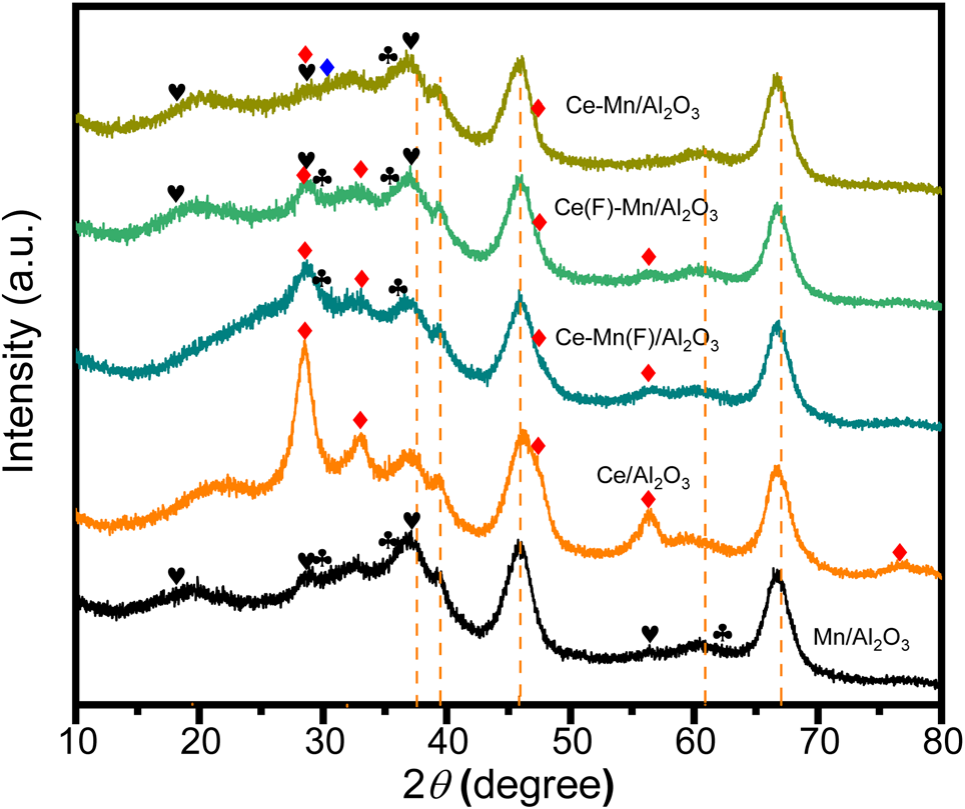
XRD patterns of Ce–Mn/Al_2_O_3_, Ce(F)–Mn/Al_2_O_3_, Ce–Mn(F)/Al_2_O_3_, Ce/Al_2_O_3_ and Mn/Al_2_O_3_.

The structural difference of Ce–Mn/Al_2_O_3_, Ce(F)–Mn/Al_2_O_3_ and Ce–Mn(F)/Al_2_O_3_ were also analyzed by XRD (Fig. 4) and the XRD patterns of Ce/Al_2_O_3_ and Mn/Al_2_O_3_ were used as a comparison. It can be seen that Ce–Mn/Al_2_O_3_ and Ce(F)–Mn/Al_2_O_3_ show similar XRD patterns to Mn/Al_2_O_3_, except that the diffraction peaks at 28.55° and 30.33° differ slightly from those of Mn/Al_2_O_3_ due to the introduction of Ce species. Both Ce–Mn/Al_2_O_3_ and Ce(F)–Mn/Al_2_O_3_ have the mixed crystal phase of MnO_2_ and Mn_3_O_4_, but the peak intensity of MnO_2_ in Ce(F)–Mn/Al_2_O_3_ is intensified. The impregnated manganese covers part of cerium and the interactions between manganese and cerium promotes the oxidation of manganese,^30^ resulting in more MnO_2_ on the Ce(F)–Mn/Al_2_O_3_ catalyst surface. In contrast, the competitive synergetic effects of cerium and manganese components promotes the surface dispersion of MnO_2_ and the CeO_2_, resulting in its weaker diffraction peak intensity. It has been reported that because the ionic radius of manganese ions (Mn^3+^: 0.066 nm) is smaller than that of Ce^4+^ (0.092 nm), manganese ions can enter into the ceria lattice, weakening the diffraction peak intensity of CeO_2_.^24,31^ As for the Ce–Mn(F)/Al_2_O_3_, the first impregnation of Mn followed by Ce has a significant effect on the crystal structure of manganese. The crystal phase of manganese in Ce–Mn(F)/Al_2_O_3_ is mainly Mn_3_O_4_ and the peak intensity of cerium oxide is also intensified. The impregnated cerium covers part of manganese and interacts with it, making it difficult to be further oxidized, mainly in the form of Mn_3_O_4_.^24,30^

#### 3.3.2 XPS analysis

Fig. S3† and 5 shows the XPS survey and high-resolution scan spectra of Ce–Mn/Al_2_O_3_, Ce–Mn(F)/Al_2_O_3_ and Ce(F)–Mn/Al_2_O_3_ catalysts, further suggesting the elemental and chemical differences of Ce, Mn and O under different impregnation sequences. Table S2† also listed the surface atomic ratios of Ce, Mn and O elements of these samples. Since the ionic radius of Ce^3+^ is larger than that of Mn^2+^, manganese is more inclined to be enriched in the pore interior than cerium, that is, the competitive impregnation occurred during the one-step impregnation. Therefore, the manganese content on the Ce–Mn/Al_2_O_3_ surface is the lowest. The two-step impregnation helps to improve the surface atomic ratio of Mn, and the Mn/Ce surface atomic ratio of Ce(F)–Mn/Al_2_O_3_ is the highest, which is close to 7 : 3.

**Fig. 5:**
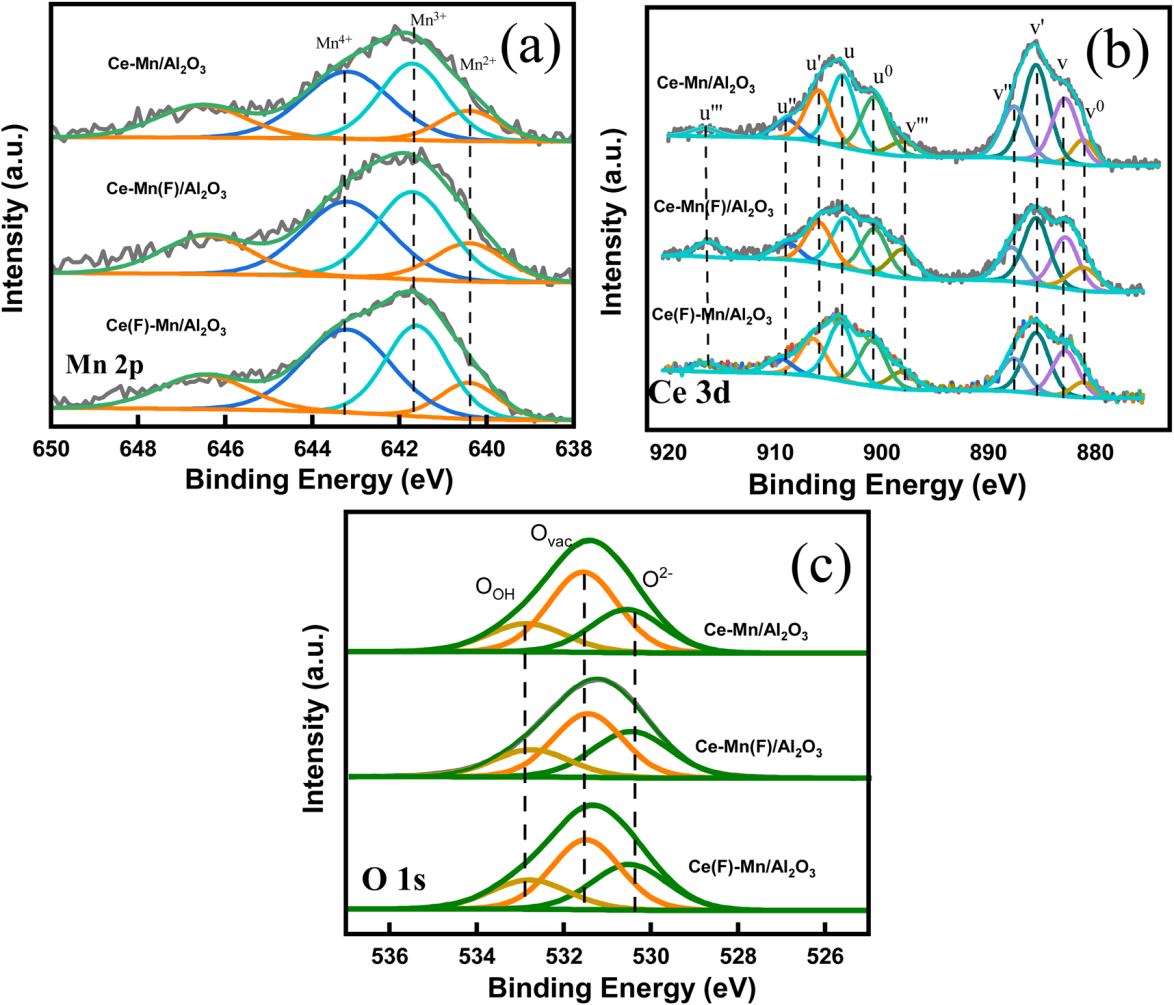
High resolution Mn 2p (a), Ce 3d (b) and O 1s (c) XPS spectra of Ce–Mn/Al_2_O_3_, Ce–Mn(F)/Al_2_O_3_ and Ce(F)–Mn/Al_2_O_3_.

Besides, the impregnation sequence has a great influence on the chemical states and relative percentage of elements on the catalyst surface (Table 1). For Mn 2p3/2 spectra, the fitting peaks at 640.4, 641.7 and 643.2 eV are ascribed to Mn^2+^, Mn^3+^ and Mn^4+^, respectively.^32–34^ The satellite peak at ∼646 eV represents the existence of Mn^2+^, and the shoulder peak at ∼643 eV represents the existence of Mn^4+^.^35–37^ As depicted in the Fig. 3(a), the ratios of Mn^4+^ to the sum of Mn^4+^, Mn^3+^ and Mn^2+^ on Ce–Mn/Al_2_O_3_, Ce–Mn(F)/Al_2_O_3_ and Ce(F)–Mn/Al_2_O_3_ are 0.38, 0.34, and 0.40. It is consistent with the XRD results that the peak intensity of MnO_2_ in Ce(F)–Mn/Al_2_O_3_ is intensified than that of Ce–Mn/Al_2_O_3._ Moreover, the ratios of Mn^2+^ are 0.30, 0.32 and 0.28, respectively. Combined with XRD analysis, the main crystal phase of manganese is Mn_3_O_4_ on Ce–Mn(F)/Al_2_O_3_, which is consistent with the increase of Mn^2+^ content in XPS results. That is, the first impregnation of Ce followed by Mn will increase the ratio of Mn^4+^, while the first impregnation of Mn followed by Ce will increase the ratio of Mn^2+^. The lowest content of Mn^4+^ on Ce–Mn(F)/Al_2_O_3_ is unfavorable for the improvement of catalytic activity.

**Table 1:** Binding energies and relative percentage of different chemical states of Mn, Ce and O of Ce–Mn/Al_2_O_3_, Ce–Mn(F)/Al_2_O_3_ and Ce(F)–Mn/Al_2_O_3_

Sample	O 1s (eV)			O_vac_/%	Mn 2p3/2 (eV)			Mn^4+^/%	Ce 3d (eV)		Ce^3+^/%
	O_OH_	O_vac_	O^2−^		Mn^4+^	Mn^3+^	Mn^2+^		Ce^4+^	Ce^3+^	
Ce–Mn/Al_2_O_3_	532.8	531.5	530.5	51.24	643.2	641.7	640.4	37.72	*uu*′′*u*′′′	*u* ^0^ *u*′	52.18
									*vv*′′*v*′′′	*v* ^0^ *v*′	
Ce–Mn(F)/Al_2_O_3_	532.8	531.5	530.5	43.75	643.2	641.7	640.4	34.49	*uu*′′*u*′′′	*u* ^0^ *u*′	45.58
									*vv*′′*v*′′′	*v* ^0^ *v*′	
Ce(F)–Mn/Al_2_O_3_	532.8	531.5	530.5	45.77	643.2	641.7	640.4	40.5	*uu*′′*u*′′′	*u* ^0^ *u*′	47.00
									*vv*′′*v*′′′	*v* ^0^ *v*′	

For the Ce 3d spectra, it consists of 5 pairs of doublets, where *u* and *v* represent the Ce 3d5/2 and 3d3/2 spin–orbit splits, respectively. The peaks *v* (882.8 eV), *v*′′ (887.5 eV), *v*′′′ (898 eV) are attributed to Ce^4+^ 3d3/2, and the peaks *u* (903.6 eV), *u*′′ (909 eV), *u*′′′ (916.5 eV) are attributed to Ce^4+^ 3d5/2. The peaks *v*^0^ (881.1 eV), *v*′ (885.5 eV) are attributed to Ce^3+^ 3d3/2, and the peaks *u*^0^ (900.7 eV), and *u*′ (906 eV) are attributed to Ce^3+^ 3d5/2.^37,38^ The ratios of Ce^3+^ to the sum of Ce^4+^ and Ce^3+^ on Ce–Mn/Al_2_O_3_, Ce–Mn(F)/Al_2_O_3_ and Ce(F)–Mn/Al_2_O_3_ are 0.52, 0.46, and 0.47, respectively. Obviously, the synergetic effects of Mn and Ce in the one-step impregnation promotes the formation of Ce^3+^, while the two-step impregnation is adverse to the formation of Ce^3+^. The presence of Ce^3+^ can greatly increase the electron donor density and promote the formation of more oxygen vacancies due to the charge compensation.^39^ By comparison, Ce–Mn/Al_2_O_3_ has the largest amount of Ce^3+^, which is conclusive to the formation of more oxygen vacancies, thereby improving its catalytic ozonation activity.^33,40^

For O 1s spectra, the fitting peaks at 530.5 eV, 531.5 eV, 532.8 eV are ascribed to lattice oxygen (O^2−^), surface atomic oxygen adsorbed on oxygen vacancies (O_vac_) and surface hydroxyl groups or chemisorbed water (O_OH_), respectively. The ratios of O_vac_ on Ce–Mn/Al_2_O_3_, Ce–Mn(F)/Al_2_O_3_ and Ce(F)–Mn/Al_2_O_3_ are 0.51, 0.44, and 0.46, respectively, indicating that there are abundant surface oxygen vacancies. It is well known that the content of oxygen vacancy in CeO_2_ catalyst is closely related to the content of Ce^3+^,^41^ and the content ordering of O_vac_ in this study is exactly consistent with that of Ce^3+^.

#### 3.3.3 EPR analysis

**Fig. 6:**
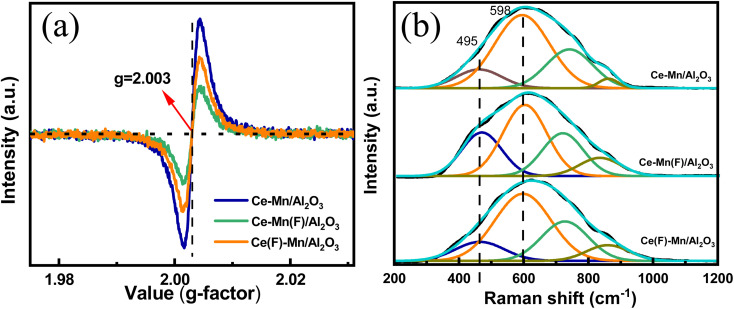
EPR (a) and Raman (b) spectra of Ce–Mn/Al_2_O_3_, Ce–Mn(F)/Al_2_O_3_ and Ce(F)–Mn/Al_2_O_3_.

To elucidate the influence of impregnation sequence on oxygen vacancy content and its subsequent impact on catalytic activity, EPR analysis was also conducted (Fig. 6(a)). A distinct signal at *g* = 2.003, characteristic of oxygen vacancy trapping electrons,^10,42^ was observed on all catalysts. CeO_2_ inherently possesses an oxygen defect structure due to its unsaturated surface atomic coordination, and the introduction of manganese further enhances the variation in Ce^3+^/Ce^4+^, thereby promoting oxygen defect formation. Specifically, the peak intensity follows the order of Ce–Mn/Al_2_O_3_ > Ce(F)–Mn/Al_2_O_3_ > Ce–Mn(F)/Al_2_O_3_, consistent with the sequence of Ce^3+^ and oxygen vacancy content (O_vac_).

#### 3.3.4 Raman analysis

Raman spectra also revealed variations in oxygen vacancies among different catalysts (Fig. 6(b)). It is known that the characteristic peak at 465 cm^−1^ corresponds to the symmetric Ce–O vibrational mode (F_2g_) of CeO_2_. In addition to this, the doped CeO_2_ also exhibits a Raman peak near 595 cm^−1^, which is attributed to the vibration peak of oxygen defects (D). All catalysts exhibited a broad band at 617 cm^−1^, which can be deconvoluted into peaks at 465, 598, 729, 869 cm^−1^, associated to F_2g_, D, and characteristic peroxide peaks.^11,43^ The ratio of peak area A_2g_/A_D_ reflects the relative abundance of oxygen vacancies. Specifically, the A_2g_/A_D_ ratios of Ce–Mn/Al_2_O_3_, Ce–Mn(F)/Al_2_O_3_ and Ce(F)–Mn/Al_2_O_3_ were 4.73, 1.80, 3.82, respectively, indicating that the concentration of oxygen vacancies followed the order of Ce–Mn/Al_2_O_3_ > Ce(F)–Mn/Al_2_O_3_ > Ce–Mn(F)/Al_2_O_3_, consistent with the EPR results.

In all, the competitive synergistic effects of cerium and manganese noticeably increased the oxygen vacancy content during the one-step impregnation process, whereas the absence of such competitive effects resulted in a decrease in oxygen vacancy content in the two-step impregnation processes.

#### 3.3.5 Comparisons of catalytic activity

**Fig. 7:**
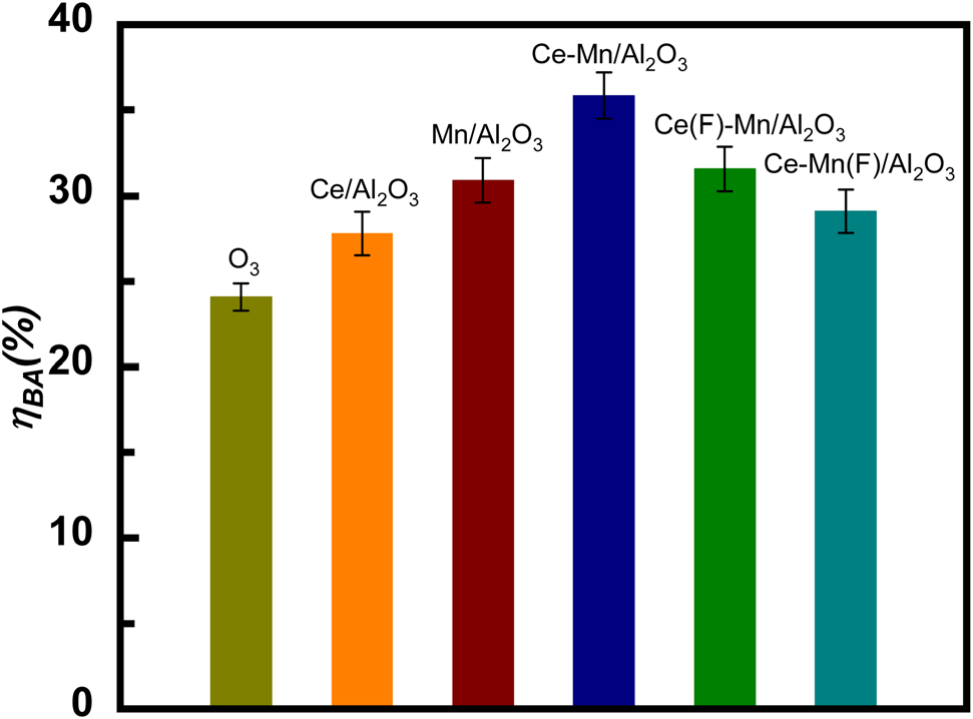
Comparison of benzoic acid degradation efficiency between single and catalytic ozonation by Ce–Mn/Al_2_O_3_, Ce–Mn(F)/Al_2_O_3_ and Ce(F)–Mn/Al_2_O_3_ (*V* = 1000 mL, *c*_BA_ = 50 mg L^−1^, *m*_s_ = 0.5 g L^−1^, *C*_O3_ = 7.5 mg L^−1^, time = 15 min).

The catalytic performance of catalysts for the ozonation of benzoic acid are shown in Fig. 7. The degradation efficiency of benzoic acid (*η*_BA_) in the catalytic ozonation by Ce/Al_2_O_3_, Mn/Al_2_O_3_, Ce–Mn/Al_2_O_3_, Ce(F)–Mn/Al_2_O_3_ and Ce–Mn(F)/Al_2_O_3_ were 27.8%, 30.9%, 35.9%, 31.6% and 29.1%, respectively, while the *η*_BA_ of the single ozonation was only 24.1% within 15 min. Under the synergetic effects of cerium and manganese, the catalytic ozonation activity of Ce–Mn/Al_2_O_3_ was greatly improved, and the *η*_BA_ of Ce–Mn/Al_2_O_3_ was 8.1% and 5.0% higher than that of Ce/Al_2_O_3_ and Mn/Al_2_O_3_, respectively. It is inferred from the XRD results that MnO_2_ plays a dominant role in the catalytic ozonation performance, and its contribution is greater than that of CeO_2_. When ceria is used as a single metal catalyst, its catalytic ozonation activity is not optimistic. Among the cerium oxides, the activity of Ce_2_O_3_ is more favorable. Therefore, the catalytic ozonation activity of Ce–Mn/Al_2_O_3_ was the highest, followed by Ce(F)–Mn/Al_2_O_3_, and then Ce–Mn(F)/Al_2_O_3._ In summary, the impregnation sequence had a great influence on the catalytic ozonation activity of catalysts, given that it can affect the synergetic effects of cerium and manganese, and then affect the metal valence state, oxygen vacancy, *etc.*

### 3.4 Catalytic ozonation activity and stability of Ce–Mn/Al_2_O_3_ on the degradation of benzoic acid

#### 3.4.1 Effects of different parameters on the catalytic ozonation degradation efficiency of benzoic acid

**Fig. 8:**
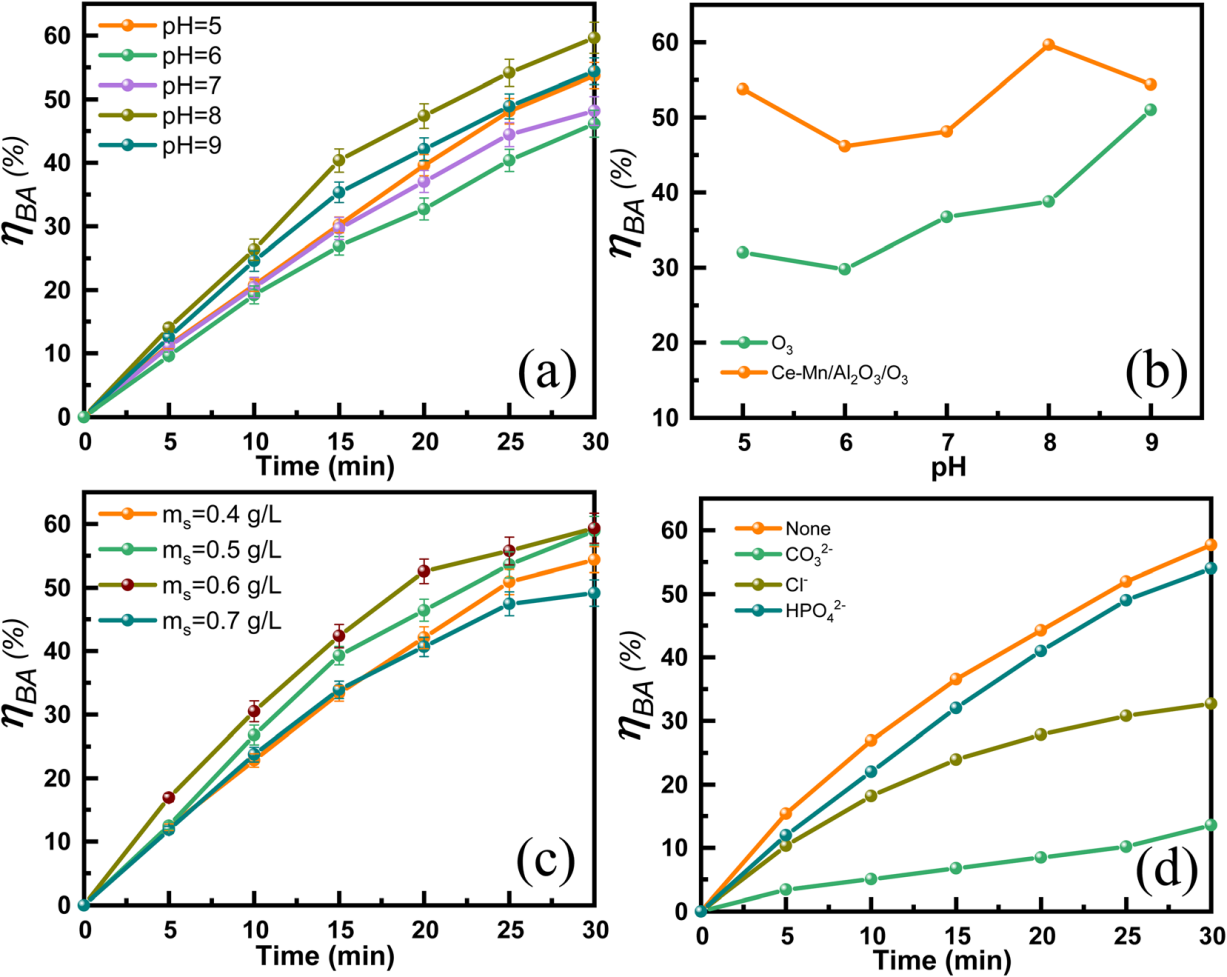
Effect of pH (a) and (b), catalyst dosage (c) and anions (d) on the catalytic ozonation degradation efficiency of benzoic acid.

Fig. 8(a) shows the effects of solution pH on the ozonation efficiency of benzoic acid catalyzed by Ce–Mn/Al_2_O_3_. The highest degradation efficiency of BA was obtained at the solution pH = 8, and the *η*_BA_ reached 59.7% within 30 min. The solution pH can affect the electrification of hydroxyl groups on the catalyst surface and the decomposition rate constant of ozone. The point of zero charge (pH_PZC_) of Ce–Mn/Al_2_O_3_ is 7.6 in this study. When the pH value is close to the pH_PZC_, the hydroxyl groups on the catalyst surface are in a neutral state, which is conducive to accelerating the dissociation of ozone to hydroxyl radical (˙OH).^44^ In addition, the alkaline condition also facilitates the initiation of ˙OH by the reaction of ozone with hydroxide ions. Thus, the *η*_BA_ increased with pH from 6 to 8 due to the enhanced indirect oxidation by the increased exposure of ˙OH. However, benzoic acid mainly exists in the anionic form when pH > p*K*_a_ (the p*K*_a_ value of benzoic acid is 4.21). When the solution pH = 9, an electrostatic repulsion will be generated between the negatively charged benzoic acid (pH > p*K*_a_) and the negatively charged Ce–Mn/Al_2_O_3_ surface hydroxyl groups (pH > pH_PZC_),^45^ thus the *η*_BA_ began to decline. When pH < pHpzc, the surface hydroxyl groups are protonated, and the positively charged oxygen is not conducive to the adsorption and dissociation of ozone to ˙OH.^44^ The degradation of BA is mainly ascribed to the direct oxidation by ozone in the acidic environment. When pH = 5, benzoic acid exists mainly in molecular form (pH ≈ p*K*_a_), which favors the nucleophilic substitution of molecular ozone, resulting in a higher *η*_BA_ at pH = 5 than at pH = 6. A similar trend was observed in single ozonation. Fig. 8(b) compares the degradation efficiency of BA under different pH values for both single ozonation and catalytic ozonation. In an acidic environment, molecular ozone attacks benzoic acid through nucleophilic substitution, resulting in the formation of phenol. As benzoic acid dissociates, the nucleophilic substitution by ozone weakens, and the degradation of BA increasingly relies on the attack by ˙OH. Consequently, starting at pH = 6, the degradation efficiency of BA in single ozonation increased with the rising pH, reaching a peak at pH = 9. This is attributed to the rapid self-decomposition of aqueous ozone into ˙OH in a strongly alkaline environment, which accelerates the degradation of BA. Overall, the degradation efficiency of catalytic ozonation is significantly higher than that of single ozonation.

Fig. 8(c) shows the effects of catalyst dosage on the catalytic ozonation efficiency of benzoic acid. The *η*_BA_ gradually increased from 54.4% to 59.3% with the catalyst dosage increases from 0.4 to 0.6 g L^−1^ due to the increased exposure of the surface reactive sites. Further increasing the catalyst dosage, the *η*_BA_ decreased. The possible reason maybe that high dosage of Ce–Mn/Al_2_O_3_ produces too much active species such as ˙OH and O_2_˙^−^, resulting in inactivation and self-quenching of active species.^45^Fig. 8(d) also shows the effects of common anions (CO_3_^2−^, Cl^−^, HPO_4_^2−^, 10 mM) on the degradation efficiency of benzoic acid. The presence of anions resulted in a significant decrease of the degradation efficiency of benzoic acid, and its inhibitory effects followed the order of CO_3_^2−^ > Cl^−^ > HPO_4_^2−^. Wang *et al.*^21^ have proposed that the dominant reactive oxygen species for the degradation of BA is ˙OH. The stepwise hydroxylation of benzoic acid is the predominant reaction pathway, during which ˙OH react with the aromatic ring to form hydroxybenzoic acids, dihydroxy-benzoic acids and trihydroxy-benzoic acids.^46^ CO_3_^2−^ is known to have a strong scavenging effect on ˙OH, with the relatively high reaction rate constant (*k* = 4.2 × 10^8^ M^−1^ s^−1^) and the *η*_BA_ significantly decreased from 57.7% to 13.6% in the presence of CO_3_^2−^. ˙OH can also be scavenged by Cl^−^ with the moderate reactivity (*k* = 8.9 × 10^7^ M^−1^ s^−1^), resulting in a 25% reduction in *η*_BA_ in the presence of Cl^−^.^47^ It is inferred that due to the relatively low reactivity of HPO_4_^2−^ with ˙OH, the *η*_BA_ was reduced by only 3.7% in the presence of HPO_4_^2−^.

#### 3.4.2 Catalytic ozonation activity of Ce–Mn/Al_2_O_3_

**Fig. 9:**
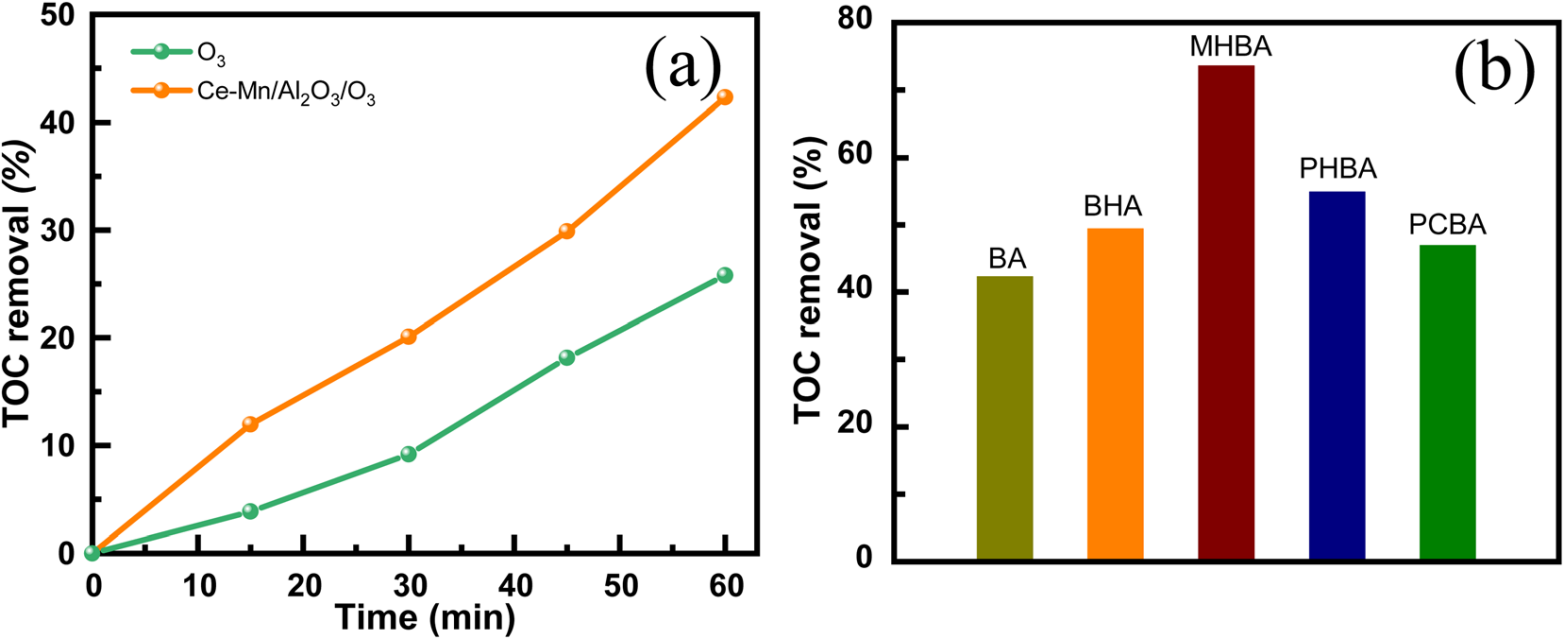
TOC removal of benzoic acid solution in the single ozonation and catalytic ozonation (a) and TOC removal of benzoic acid derivatives during the catalytic ozonation (b) (*V* = 1000 mL, *c*_0_ = 50 mg L^−1^, *m*_s_ = 0.5 g L^−1^, *C*_O_3__ = 7.5 mg L^−1^, time = 60 min).

Fig. 9(a) illustrates the TOC removal efficiency of benzoic acid solution in single ozonation and catalytic ozonation. The single ozonation removed only 25.8% of TOC within 60 min, whereas the catalytic ozonation by Ce–Mn/Al_2_O_3_ removed 42.3% of TOC from the benzoic acid solution, confirming the high ozonation activity of Ce–Mn/Al_2_O_3_. To further evaluate the ozonation activity of Ce–Mn/Al_2_O_3_ on refractory pollutants, we investigated its catalytic ozonation performance on representative benzoic acid derivatives, including salicylic acid (BHA), *m*-hydroxybenzoic acid (MHBA), *p*-hydroxybenzoic acid (PHBA), and *p*-chlorobenzoic acid (PCBA). As shown in Fig. 9(b), Ce–Mn/Al_2_O_3_ exhibited greater catalytic activity towards these benzoic acid derivatives, with the TOC removal efficiency following this order: MHBA > PHBA > BHA > PCBA > BA. Notably, the catalytic ozonation with Ce–Mn/Al_2_O_3_ removed 73.6% of TOC from the MHBA solution, and the TOC removal efficiency increased significantly with the introduction of the substituted group. Previous study has demonstrated that the introduction of –OH group on the benzene ring can change the stability of benzene ring and the reactivity with ozone due to the electron-donating effect, making it more prone to ring-opening reaction.^21^ In the case of BHA, the *ortho*-effect appears to hinder the nucleophilic substitution of molecular ozone, resulting in poorer decarboxylation compared to other –OH substitutions. Conversely, the *meta*-substitution in MHBA enhances nucleophilic addition by ˙OH and facilitates stepwise hydroxylation, promoting ring-opening reactions. For the substituted –Cl group, the electron-withdrawing effect also weakens the attack of molecular ozone on –COO^−^ group, with the observed TOC removal efficiency primarily resulting from the attack of ˙OH.^21,46^

Table 2 presents a comparison of the treatment efficiency of catalytic ozonation of BA with previous studies. The catalytic performance of Ce–Mn/Al_2_O_3_ in this study is at a moderate level compared with other reported catalysts, primarily due to the low ozone concentration and the large volume of the BA solution used. Notably, the spherical shape of the Ce–Mn/Al_2_O_3_ catalyst facilitates easier recovery from the aqueous solution compared to other powder-form catalysts.

**Table 2:** Comparison of treatment efficiency with previous studies

Catalyst	*C*_O_3__	*m*_s_	*c*_BA_/*V*_BA_	Time	TOC removal efficiency	Ref.
CeO_2_ film	15 mg min^−1^	3 mg L^−1^	115 mg L^−1^/0.4 L	120 min	84%	20
Fe-shaving	60.8 mg min^−1^	33.3 g L^−1^	122 mg L^−1^/1.5 L	90 min	56%	21
Mn/ZSM	20 mg min^−1^	0.5 g L^−1^	67.5 mg L^−1^/0.5 L	30 min	75.4%	46
CeO_*x*_/AC	—	0.5 g L^−1^	50 mg L^−1^/0.3 L	30 min	35%	18
CePO_4_/CeO_2_	—	0.2 g L^−1^	2.9 mg L^−1^/0.2 L	60 min	40%	19
Ce–Mn/Al_2_O_3_	3.75 mg min^−1^	0.5 g L^−1^	50 mg L^−1^/1 L	60 min	42.3%	This study

#### 3.4.3 Reusability and stability of Ce–Mn/Al_2_O_3_

**Fig. 10:**
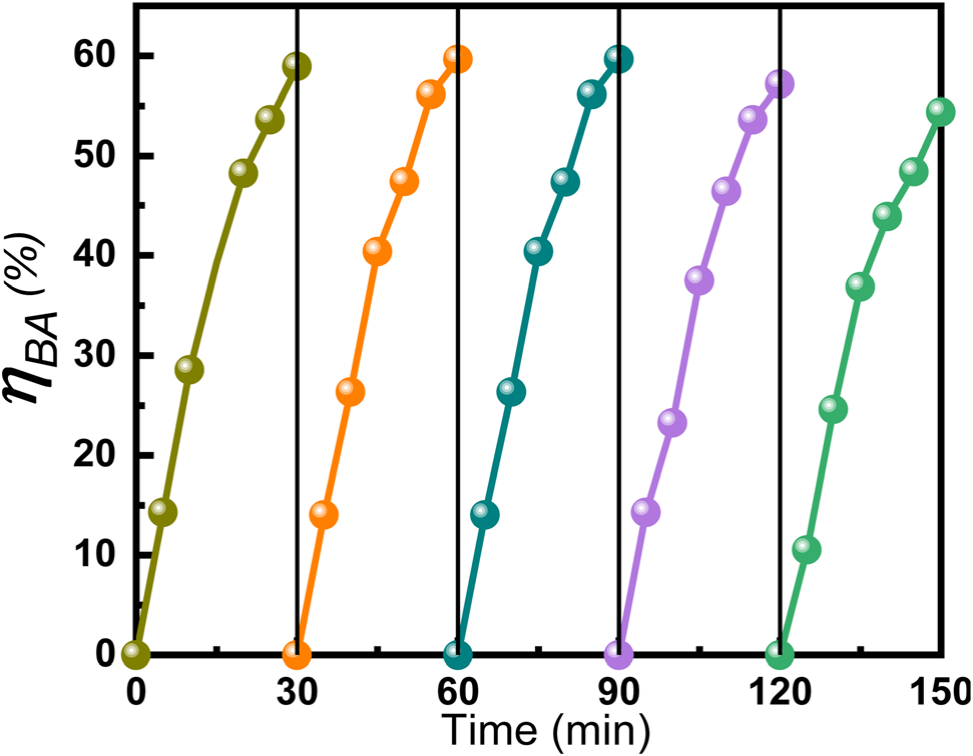
The ozonation degradation efficiency of benzoic acid catalyzed by the reused Ce–Mn/Al_2_O_3_.

**Fig. 11:**
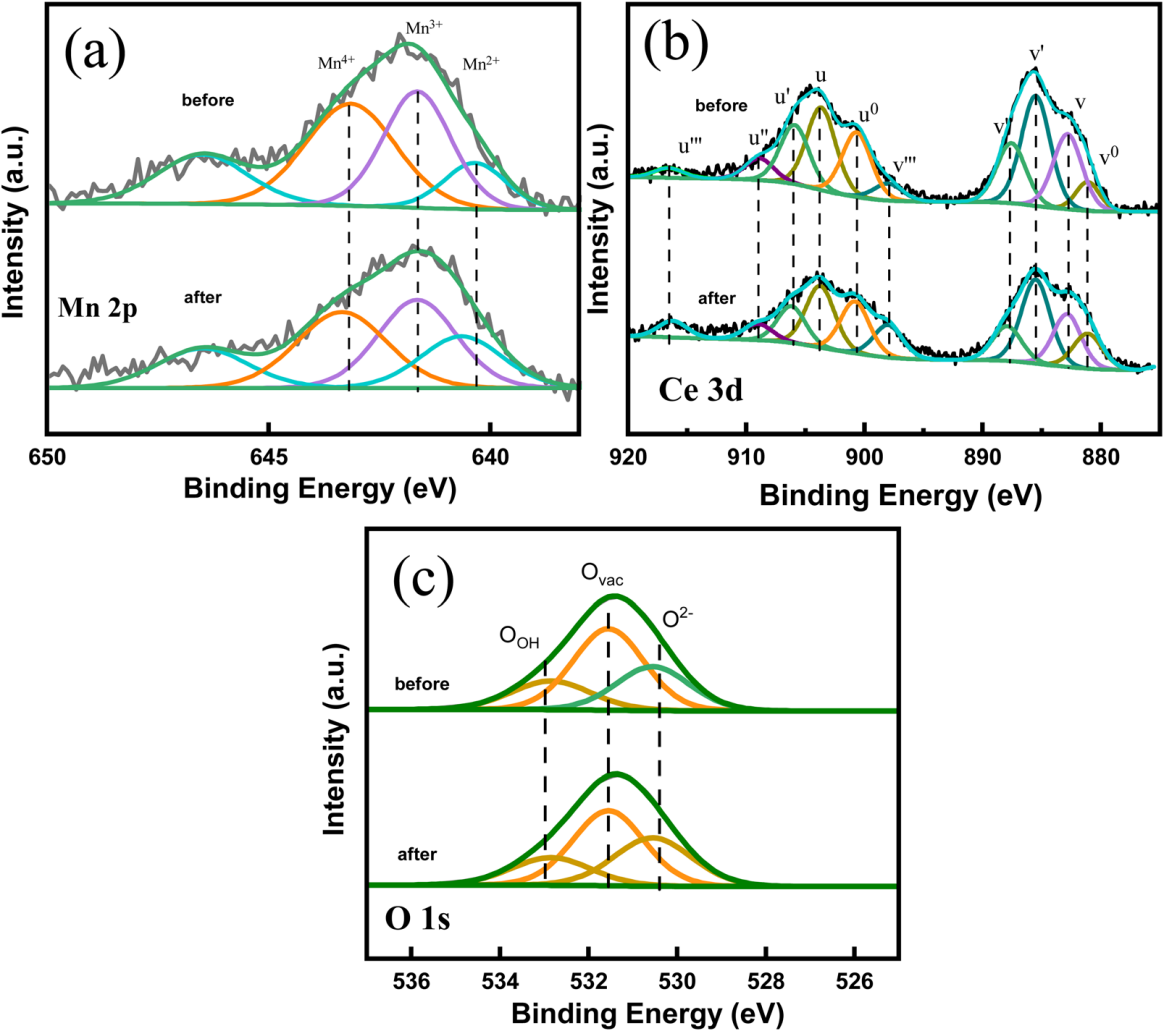
High resolution Mn 2p (a), Ce 3d (b) and O 1s (c) XPS spectra of Ce–Mn/Al_2_O_3_ before and after use.

The reusability of Ce–Mn/Al_2_O_3_ was investigated through the catalytic ozonation of benzoic acid by recycling Ce–Mn/Al_2_O_3_ catalysts and the results were shown in Fig. 10. The *η*_BA_ remained stable and increased slightly in the first three cycles due to the increased accessibility of surface-active sites. From the 4th cycle, the *η*_BA_ began to decline and decreased from 58.9% to 54.4% until the 5th cycle. The slight decrease in the catalytic ozonation activity of Ce–Mn/Al_2_O_3_ may be caused by the deactivation of the active sites due to the oxidation of ozone and reactive oxygen species, or the leaching loss of metal ions. Therefore, we also used XPS to analyze the chemical states and relative percentages of elements on the surface of Ce–Mn/Al_2_O_3_ after the fifth use (Fig. 11).

As shown in Table 3, the ratio of Mn^4+^ to the sum of Mn species decreases from 37.7% to 36.3%, while the ratio of Mn^3+^ increases from 32.2% to 36.5% after catalytic ozonation. The ratio of Ce^3+^ to the sum of Ce species decreases from 52.2% to 45.7% and the ratio of Ce^4+^ increases from 47.8% to 54.3%. The reduction of Mn^4+^ and the oxidation of Ce^3+^ occurred simultaneously in the catalytic ozonation process.^10,48–50^ The significant increase in Ce^4+^ might result in an increase in the lattice oxygen, which is converted by the oxygen uptake at oxygen vacancies. Ozone molecule can also be adsorbed on the oxygen vacancy to form superoxide anion radicals,^51^ and the surface hydroxyl groups can also be attacked by ozone molecule to form ozonide anion radicals.^52^ As a result, the ratio of O_vac_ decreases from 51.2% to 49.9% and the ratio of O_ads_ also decreases from 20.3% to 18.2% after catalytic ozonation. In contrast, the ratio of O^2−^ increases from 28.5% to 31.9%. These results indicates that the active centers of catalytic ozonation of benzoic acid are mainly Mn^4+^, Ce^3+^, surface hydroxyl groups and oxygen vacancies. Although the inactivation and loss of the active sites occurred during the ozonation process, the synergetic interactions between cerium and manganese atoms enable the rapid electron transfer between Ce^4+^ and Mn^3+^ to promote the recovery of Ce^3+^ and Mn^4+^:^53^

2 Ce^4+^ + Mn^3+^→ Mn^4+^ + Ce^3+^

**Table 3:** Binding energies and relative percentage of different chemical states of Mn, Ce and O of Ce–Mn/Al_2_O_3_ before and after use

Sample	O 1s (eV)			O_vac_/%	Mn 2p3/2 (eV)			Mn^4+^/%	Ce 3d (eV)		Ce^3+^/%
	O_ads_	O_vac_	O^2−^		Mn^4+^	Mn^3+^	Mn^2+^		Ce^4+^	Ce^3+^	
Before	532.8	531.5	530.5	51.24	643.2	641.7	640.4	37.72	*uu*′′*u*′′′	*u* ^0^ *u*′	52.18
									*vv*′′*v*′′′	*v* ^0^ *v*′	
After	532.6	531.5	530.4	49.87	643.4	641.7	640.7	36.30	*uu*′′*u*′′′	*u* ^0^ *u*′	45.66
									*vv*′′*v*′′′	*v* ^0^ *v*′	

To illustrate this, we also studied the adsorption configuration of ozone on the active centers of Ce–Mn/Al_2_O_3_. The γ-Al_2_O_3_ structure is established and optimized according to the cell parameters provided by Digne *et al.*^54^ The cell parameters are *a* = 5.59 Å, *b* = 8.42 Å, *c* = 8.10 Å and *β* = 90.60°. Since the (110) surface is considered to be the most exposed crystal surface of γ-Al_2_O_3_ (70–83%), the (110) surface is chosen as the representative of γ-Al_2_O_3_. The Ce–Mn/Al_2_O_3_ model is established by adsorption of the optimized Ce–Mn cluster on the surface of Al_2_O_3_(110). After optimization, Mn atom is bonded to O60, O61 and O62 with bond lengths of 2.30 Å, 1.90 Å and 2.04 Å respectively, and Ce atom is bonded to O23 and O15 with bond lengths of 2.38 Å, as shown in Fig. S4.†

**Fig. 12:**
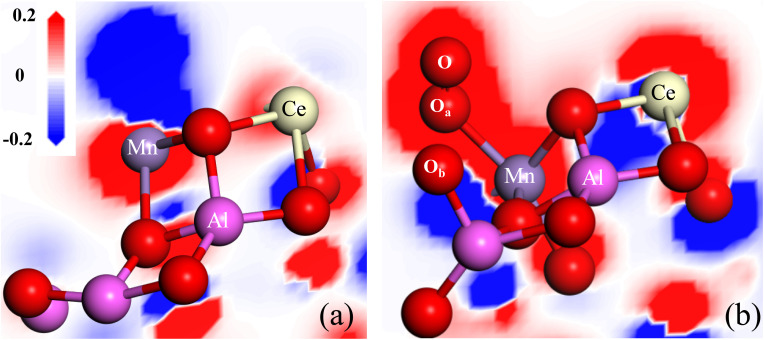
The differential charge density adjacent to the oxygen vacancy of Ce–Mn/Al_2_O_3_(110) surface before (a) and after (b) ozone adsorption.

The unsaturated coordination oxygen O64 can easily transfer electrons to Mn atom and release oxygen to form oxygen vacancy, given that its Mulliken charge is −0.364|*e*| and its conformation is far away from the Ce–Mn/Al_2_O_3_(110) surface. The calculated oxygen vacancy formation energy *E*_OV_ is only 0.02 eV, indicating that the (110) surface of Ce–Mn/Al_2_O_3_ is very active to form oxygen vacancy defects. As shown in Fig. 12(a), with the formation of oxygen vacancy, the charge density around the oxygen vacancy drops sharply, resulting in a significant increase in the charge density around the Mn atom, forming an electron-rich center to maintain electrical neutrality. Meanwhile, there is also electron transfer between Mn and Ce atoms, and the charge density around Ce atom increase slightly due to the dual redox reaction of Mn^3+^/Mn^4+^ and Ce^4+^/Ce^3+^.

When the ozone molecule is adsorbed on the oxygen vacancy of Ce–Mn/Al_2_O_3_(110) surface, it can be dissociated into *O_2_ and bonded to the Mn atom. The O–O a bond length is 1.26 Å, which is exactly the same as that of O_2_˙^−^. At the same time, an *O atom is also formed and bonded to the Al atom (Fig. 12(b)), thus replenishing the released lattice oxygen. The dissociation energy of ozone on the oxygen vacancy of Ce–Mn/Al_2_O_3_(110) surface is −34.18 kcal mol^−1^, and the reaction energy barrier is only 1.616 kcal mol^−1^. This process is very favorable both thermodynamically and kinetically. With the adsorption and dissociation of ozone on the oxygen vacancy, the charge density around Mn atom increases, while the charge density around Ce atom decreases, indicating that the adsorption of ozone on the oxygen vacancy is accompanied by the reduction of Mn^4+^ and the oxidation of Ce^3+^. In a word, the oxygen vacancy acts as an effective active site for ozone adsorption and dissociation into *O_2_ and *O, so the ratio of O_ads_ increases correspondingly after catalytic ozonation, and this process also involves the dual redox reactions of Mn^4+^/Mn^3+^ and Ce^3+^/Ce^4+^ to form an electron closed loop and to maintain the balance of electron supply, which fully explains and supports our previous experimental results.

## 4. Conclusion

Ce–Mn binary oxides supported on Al_2_O_3_ with enhanced activity and stability for catalytic ozonation of benzoic acid have been obtained by one-step impregnation method. The different impregnation conditions especially impregnation sequence have a great influence on the structure and the catalytic ozonation performance of catalysts. The diffraction peaks of Ce_2_O_3_ and MnO_2_ appeared in the catalyst with Mn/Ce = 7 : 3, and the calcination temperature of 600 °C enhanced the intensity of Ce_2_O_3_, which are favorable to the improvement of catalytic ozonation activity. Ce–Mn/Al_2_O_3_ demonstrated strong catalytic ozonation activity towards benzoic acid derivatives, achieving a TOC removal of 42.3% from benzoic acid and 73.6% from *m*-hydroxybenzoic acid solution within 60 min. The competitive synergetic effects of cerium and manganese in the one-step impregnation process promoted the dispersion of Ce component, resulting in more homogeneous Ce^3+^ species on the catalysts surface, which substantially increased the surface oxygen vacancies of catalysts, while the lack of competitive effects in the two-step impregnation process led to a decrease in oxygen vacancy content. Oxygen vacancies act as the effective active sites for ozone adsorption and dissociation to *O_2_ and *O, accompanied with the reduction of Mn^4+^ to Mn^3+^ and the oxidation of Ce^3+^ to Ce^4+^, to form an electron closed loop and maintain the balance of electron supply. The synergetic interactions between cerium and manganese also enable the rapid electron transfer between Ce^4+^ and Mn^3+^ to promote the recovery of Ce^3+^ and Mn^4+^. Due to the synergetic effects, the catalytic ozonation activity and stability of Ce–Mn/Al_2_O_3_ was enhanced.

## Data availability

The data supporting this article have been included as part of the ESI.†

## Conflicts of interest

The authors declare no competing financial interest, and manuscript is approved by all authors for publication.

## Supplementary Material

RA-014-D4RA06148A-s001
