# Supplementary material for: Effect of impregnation strategy on structural characteristics of Ce–Mn/Al2O3 and its catalytic ozonation of benzoic acid
Source: RSC Adv. 2024 Sep 30;14(42):30990–1002. doi: 10.1039/d4ra06148a (PMC11440235; doi:10.1039/d4ra06148a)
Supplement: RA-014-D4RA06148A-s001 [file RA-014-D4RA06148A-s001.pdf]

Supplementary Information (SI) for RSC Advances.
This journal is © The Royal Society of Chemistry 2024

Effect of impregnation strategy on structural characteristics of Ce-Mn/Al_2O_3 and its catalytic ozonation of benzoic acid

Shengjuan Shao [a*], *Ting Cheng* [b], *Yifan Cheng* [a], *Bingxin Chen* [a]

[a]. *Department of Chemistry and Chemical Engineering, Taiyuan Institute of Technology, Taiyuan, 030008, China*

[b]. *School of Chemistry and Chemical Engineering, North University of China, Taiyuan, Shanxi 030051, China*

*Corresponding author

E-mail: shaosj_tit@163.com

Figures and Tables

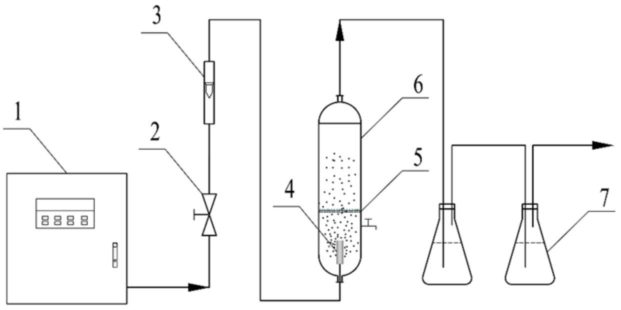

1-ozone generator；2-valve；3-gas flow meter；4-bubble aerator；5-porous separator；6-column reactor；7-KI absorbent

Figure S1 Process flow diagram of heterogeneous catalytic ozonation of benzoic acid wastewater

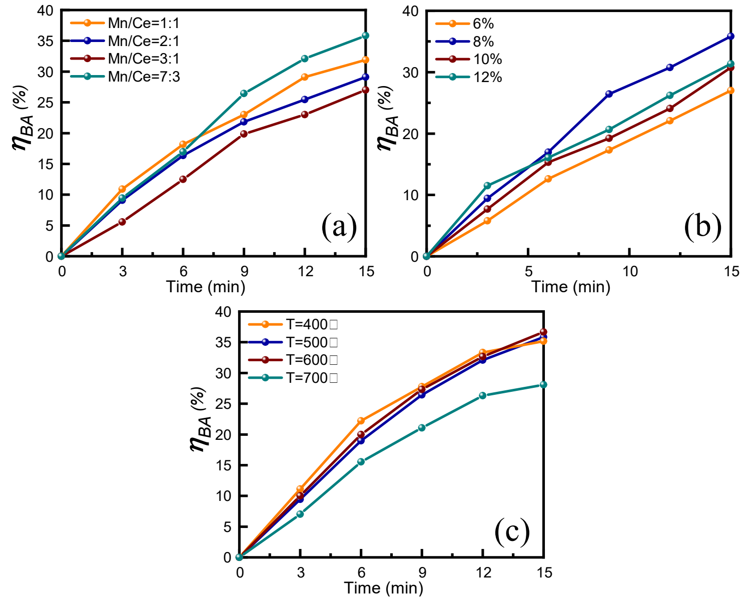

Figure S2 Effect of catalyst preparation parameters on catalytic activity of Ce-Mn/ Al_2O_3: (a) the

effects of different Mn to Ce molar ratios (Mn/Ce); (b) the effects of different metal loadings, (c) the effects of different calcination temperature (T). ($[BA]_0$= 50 mg L^{-1}, V_L = 1000 mL, m_s = 0.5 g L^{-1}, V_{O3} = 0.5 L min^{-1}, C_{O3} = 7.5 mg L^{-1})

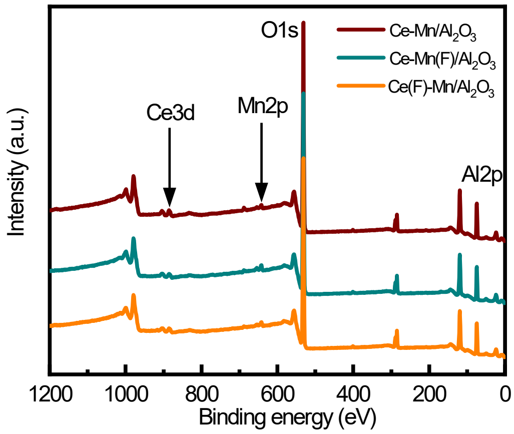

Figure S3 The XPS survey spectra of Ce-Mn/Al_2O_3, Ce-Mn(F)/Al_2O_3 and Ce(F)-Mn/Al_2O_3.

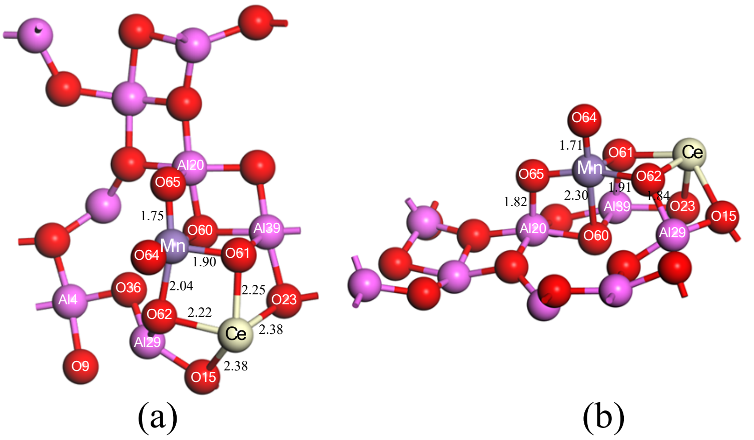

Figure S4 Top view (a) and side view (b) of Ce-Mn/Al_2O_3 (110) surface

Table S1 BET surface area, total pore volume and average pore size of Ce-Mn/Al_2O_3 with different metal loadings.

Metal loadings (wt.)	S_{BET} (m^2/g)	V_p (cm^3/g)	D_a (nm)
6%	218.41	0.74	12.59
8%	217.07	0.67	12.34
10%	216.57	0.67	12.45
12%	220.00	0.67	12.22
Al_2O_3	249.49	0.81	9.55

Table S2 Al, Mn, Ce and O contents of Ce-Mn/Al_2O_3, Ce-Mn(F)/Al_2O_3 and Ce(F)-Mn/ Al_2O_3 by XPS.

Catalyst	Al (wt. %)	Mn (wt. %)/(at. %)	Ce (wt. %)/(at. %)	O (wt. %)
Ce-Mn/ Al_2O_3	44.35	1.72/0.65	3.75/0.55	50.17

Ce-Mn(F)/Al_2O_3	44.71	2.05/0.77	3.47/0.51	49.76
Ce(F)-Mn/ Al_2O_3	44.52	2.48/0.93	3.07/0.45	49.93

Table S3 Al, Mn, Ce and O contents of Ce-Mn/Al_2O_3 before and after ozonation by XPS.

Catalyst	Al (wt. %)	Mn (wt. %)	Ce (wt. %)	O (wt. %)
before	44.35	1.72	3.75	50.17
after	44.73	1.42	3.07	50.78

Original data of Figure 8(a)

Time(min)	$\eta_{pH=5}$	$\eta_{pH=6}$	$\eta_{pH=7}$	$\eta_{pH=8}$	$\eta_{pH=9}$
5	11.32±0.56	9.61±0.54	11.11±0.65	14.04±0.62	12.53±0.58
10	20.75±0.96	19.23±1.42	20.37±1.6	26.32±1.65	24.56±1.62
15	30.19±1.24	26.92±1.44	29.62±1.76	40.35±1.84	35.33±1.64
20	39.62±1.7	32.69±1.72	37.03±1.72	47.37±1.92	42.11±1.8
25	48.11±2	40.38±1.76	44.44±1.88	54.14±2.12	48.88±1.96
30	53.72±2.04	46.15±2.1	48.14±2.26	59.65±2.46	54.39±2.08

Original data of Figure 8(b)

Time(min)	$\eta_{ms=0.4}$	$\eta_{ms=0.5}$	$\eta_{ms=0.6}$	$\eta_{ms=0.7}$
5	12.28±0.53	12.5±0.57	16.95±0.65	11.86±0.55
10	22.81±1.06	26.79±1.53	30.51±1.63	23.73±1.15
15	33.33±1.22	39.29±1.44	42.37±1.84	33.9±1.36
20	42.11±1.74	46.43±1.72	52.54±1.91	40.68±1.52
25	50.88±1.97	53.57±2.02	55.74±2.18	47.46±1.88
30	54.39±2.06	58.93±2.26	59.32±2.35	49.15±2.06

Original data of Figure 8(c)

Time(min)	η	η_{co3}	η_{cl}	η_{HPO4}
5	15.38	3.39	10.31	12
10	26.92	5.08	18.15	22
15	36.54	6.78	23.92	32
20	44.23	8.47	27.85	41
25	51.92	10.17	30.77	49

30	57.69	13.56	32.69	54

Original data of Figure 9

Time(min)	*1st run*	*2nd run*	*3rd run*	*4th run*	*5th run*
5	14.28	14.04	14.03	14.28	10.52
10	28.57	26.32	26.31	23.21	24.56
15	39.28	40.35	40.35	37.5	36.84
20	48.21	47.37	47.36	46.42	43.85
25	53.57	56.14	56.140	53.57	48.36
30	58.92	59.65	59.64	57.14	54.38
